# Lipid Profiles Alter from Pro-Atherogenic into Less Atherogenic and Proinflammatory in Juvenile Idiopathic Arthritis Patients Responding to Anti TNF-α Treatment

**DOI:** 10.1371/journal.pone.0090757

**Published:** 2014-03-06

**Authors:** Kuo-Wei Yeh, Chi-Ming Lee, Chee-Jen Chang, Yu-Jr Lin, Jing-Long Huang

**Affiliations:** 1 Division of Allergy, Asthma and Rheumatology, Department of Pediatrics, Chang Gung Memorial Hospital at Linkou, and Chang Gung University, Taoyuan, Taiwan; 2 Graduate Institute of Clinical Medical Science, Chang Gung University, Taoyuan, Taiwan; 3 Biostatistical Center for Clinical Research, Chang Gung Memorial Hospital at Linkuo, Taoyuan, Taiwan; University of Leicester, United Kingdom

## Abstract

**Objective:**

Dyslipidemia with higher inflammatory states, disease activity, and longer disease duration in juvenile idiopathic arthritis (JIA) patients seemed to increase the risks of atherosclerosis. Tumor necrosis factor- α (TNF-α) receptor blocking agent etanercept has been proven to be effective in JIA. However, data about the correlation of anti-inflammatory treatment on lipid profiles and atherogenic index in JIA patients remains limited. This study aimed to investigate the longitudinal changes on lipid profiles and atherogenic index in JIA patients after etanercept treatment.

**Methods:**

Twenty-three patients diagnosed with JIA (polyarticular type n = 7; oligoarticular type, n = 2; systemic type, n = 10, Enthesitis-related arthritis = 4) received treatment with etanercept during the period 2004–012 in a medical center. We measured their serum lipid profiles at baseline and 2, 4, 6, 12 months later, and determined whether there were differences in complete blood counts, inflammatory mediators, lipid levels and atherogenic indices between patients who had inactive disease (responders) and those who were poor responders (non-responders) to etanercept treatment.

**Results:**

Analysis of dynamic change in total JIA patients before and after TNF inhibitor therapy showed modest increases in hemoglobin levels (P = 0.02) and decreases in WBC counts, Platelet and CRP levels progressively (p = 0.002, p = 0.006 and p = 0.006, respectively).Twelve of the 23 patients achieved inactive disease status (responders) after 12-months of treatment. In responders, compared to non-responders, total cholesterol (TC), low-density lipoprotein cholesterol (LDL-C) and high-density lipoprotein cholesterol (HDL-C) increased significantly (P = 0.007,P = 0.044,P<0.001), whereas triglyceride and atherogenic index (TC/HDL-C ratio) significantly decreased (P = 0.04, P = 0.01, respectively) after etanercept treatment.

**Conclusion:**

Disease severity was associated with triglyceride level, atherogenic index and was inversely associated with total cholesterol, HDL-C, and LDL-C levels and can be improved substantially by using anti TNF-α treatment. Such treatment may have a beneficial effect on the cardiovascular risk in patients with JIA.

## Introduction

Chronic inflammatory diseases such as rheumatoid arthritis, systemic lupus erythematosus had been proven to have a higher risk of premature coronary artery disease [1]. Abnormal lipoprotein levels play an important role in atherosclerotic processes that can be related to autoimmune disease. The risk to develop atherosclerosis increases progressively with increasing low-density lipoprotein cholesterol (LDL-C) and hypertriglyceridemia levels and declines with increased levels of high-density lipoprotein cholesterol (HDL-C) [2], [3]. In adult patients with rheumatoid arthritis, cardiovascular disease is the leading cause of shortened life expectancy relative to the general population, and nearly half of these deaths can be attributed to cardiovascular disease that is linked to inflammation and elevated C-reactive protein (CRP) levels [4]. However, data regarding dyslipidemia prevalence and related impact are seldom seen and do not conclusively define the role of JIA in this metabolic disturbance.

JIA is the most common rheumatic disease in childhood, and represents a major cause of functional disability in children. JIA is also a heterogeneous and multi-factorial autoimmune disease characterized by chronic joint inflammation [5]–[7]. In JIA, studies have demonstrated an imbalance favoring the production of pro-inflammatory cytokines, including interleukin-1β (IL-1β), interleukin-6 (IL-6), and tumor necrosis factor-alpha (TNF-α), that are important contributors to the perpetuation of the inflammatory response [8]. Non-steroidal anti-inflammatory drug, methotrexate and glucocorticoid are the standard and first line treatment regimen for JIA [9]. Such traditional therapy is not always effective and has unknown toxic side effects. Most patients with systemic or polyarticular-onset JIA need other second-line medications. Etanercept, is a soluble fusion protein comprised of the extracellular domain of the TNF receptor (p75) and Fc portion of human immunoglobulin G1, and is the drug of choice for disease-modifying antirheumatic drugs refractory RA [10], [11]. It also has a beneficial effect in patients with JIA that had previously had no response or were refractory to conventional therapy [12], [13].

Disease activity and inflammatory status are inversely correlated with changes in plasma total and HDL cholesterol levels and positively correlated with the variation of atherogenic index in RA patients after anti-TNF therapy [14]. Dyslipidemia was also observed in JIA patients with higher disease activity, and longer disease duration seemed to increase the risks of atherosclerosis [15]. However, the correlation of lipid profile changes and disease activity before and after anti-TNF therapy has seldom been examined. To clarify the relationship between disease activity and the dynamic changes of complete blood counts, inflammatory status, and lipid profile in JIA, we undertook a longitudinal study to investigate serum lipid levels and atherogenic index, as well as their association with the clinical and laboratory parameters of disease activity in JIA patients.

## Materials and Methods

Data were gathered from the pediatric rheumatologic clinic of the Chang Gung Memorial Hospital, a tertiary teaching medical center in Taiwan.This study was approved by the Institutional Review Board of the Chang Gung Memorial Hospital. No additional financial support from pharmaceutical company was accepted to perform the study.

We selected patients fulfilling the International League of Associations for Rheumatology -derived criteria for JIA from July 2006 to October 2012 [16]. The patients were categorized according to JIA onset type, defining of classification took place during the first 6 months of disease [9], [16]. All patients were seen and followed up regularly by their pediatric rheumatologist (JLH) monthly. In this analysis, we excluded patients with relevant comorbidities (such as familial hypercholesterolemia, cholestatic liver disease, diabetes mellitus, nephrotic syndrome, thyroid disease, Cushing syndrome or obesity (body mass index >30 kg/m^2^) that affect lipid profiles or took drugs such as lipid-lowering drugs, beta-blockers, oral contraceptives, thyroxin during the study period.

After 12 months of etanercept treatment, inactive disease(responders to anti-TNF therapy treatment) was defined, according to a modified definition of Wallace [17] that involved no active arthritis, no fever, rash, serositis, splenomegaly, or generalized lymphadenopathy attributed to JIA; no active uveitis; normal erythrocyte sedimentation rate or C-reactive protein (CRP) level; and a physician' global assessment of disease activity indicating no disease activity. Active disease (non-responders to anti-TNF therapy treatment) was divided into two subtypes. In systemic arthritis, it was defined as at least 1 joint with active arthritis or fever of ≥38.5°C at least 4 days per week without definable infection or other cause. In oligoarthritis, polyarthritis and enthesitis-related arthritis, it was defined as at least 1 joint with active arthritis with swelling not due to bony enlargement or, if no swelling was present, limitation of motion accompanied by either pain on motion and/or tenderness. All patients showed active disease in the beginning and treated with either concomitant medication, including methotrexate and low-dose prednisolone, or etanercept monotherapy during the followed-up period. The dose of etanercept was 0.4 mg/kg, with maximal 25 mg, subcutaneously, twice a week. This final cut-off time was defined after 12 months etanercept treatment. Because all good responders developed inactive disease for more than 6 months and fulfilled the Wallace's criteria of the clinical remission [17].

### Blood sampling and laboratory monitoring

Overnight fasting blood samples were obtained 1 month before etanercept treatment (baseline) and at 2, 4, 6, and 12 months after start of the treatment. Serum total cholesterol, triglyceride and high-density lipoprotein cholesterol were analyzed by an enzymatic colorimetric method (Hitachi 7600, Hitachi, Tokyo, Japan). Serum total cholesterol and triglyceride were analyzed by an enzymatic method using the appropriate assays (Roche Diagnostics, Almere, The Netherlands) on a Cobas 6000 analyzer (Roche Diagnostics) according to the manufacturer's instructions. The Friedewald formula was estimated to calculate LDL-C [18]. When triglyceride levels were lower than 400 mg/dl (4.5 mmol/L), the TC to HDL-C and LDL-C (atherogenic index) were calculated [19], [20]. Human leukocyte antigen HLA-B27 antigen (by microlymphocytotoxicity assay), antinuclear antibodies (ANA; by indirect immunofluorescence assay) and rheumatoid factor (RF; by nephelometry) were analyzed at onset. C-relative protein (CRP), complete blood counts (including total white blood cell counts, platelet counts, and hemoglobin levels) were calculated by nephelometry. The values of rheumatoid factor ≥15 IU/ml or the titers of antinuclear antibodies ≥1∶40 were classified as positive. The hospital's Institutional Review Board approved the study and all participants and/or their parents provided written informed consent.

### Statistical analysis

To determine the effects of etanercept, treatment with JIA disease activity, we divided the JIA patients into active (non-responders) and inactive (responders) groups based on the disease activity evaluated at 12 months. Significant differences in levels of CRP, complete blood counts and lipid profile at baseline and after treatment with etanercept were tested by a paired-sample t test. In a first data analysis, generalized estimating equation (GEE) regression model to compare active and inactive group differences was used to analyze longitudinal data at five different time points (baseline, 2,4,6 and 12 months). Secondly, we adjusted one at a time for the additional covariates age, and gender in order to test whether responder status was an effective modifier for specific variables, and interaction terms. Statistical analyses were performed using the SPSS software package for Windows (version 17.0; SPSS, Chicago, IL) and p value of less than 0.05 was considered statistically significant.

## Results

### Characteristics of the patients at baseline and changes in disease activity

Among the 23 children (10 boys and 13 girls), 7 patients had polyarticular type of JIA, 2 patients had oligoarthritis of JIA (persistent type), 4 patients had enthesitis-related arthritis and 10 patients had systemic onset of JIA. Anti-nuclear antibody was positive in twelve patients (52.1%, 12/23) including five patients presented with both Rheumatoid factor and ANA positive (21.7%, 5/23). Human leukocyte antigen-B27 was positive in seven patients (30.4%, 7/23) and two of them also with measurable ANA. The mean age at diagnosis of JIA was 8.05 (3.4–15.0) years, and the mean age of patients using etanercept was 11.9 (4.1–19.4) years. Twelve patients (52.1%) were classified into the inactive group (responders) according to the disease activity evaluated at 12 months, and eleven patients (47%) were classified into the active group (non-responders) (Table 1). Prednisolone was used in the beginning and then tapered and discontinued at a mean of 2.02months (range, 1–4months) in responders after starting etanercept treatment. In non-responders, they kept using methotrexate and/or prednisolone because of poor responses or flare up to etanercept therapy. There were no adverse effects such as atypical mycobacteria infection, sepsis, or cellulitis during the follow-up period.

**Table 1:** Baseline characteristics and treatment of patients with diagnosed JIA, stratified by their disease activity at 12 months*.

	Active JIA (n = 11) (non-responders)	Inactive JIA (n = 12) (responders)
Age of diagnosis	9.05±4.68	8.01±4.18
Female	8(72.7)	6 (50)
RF positive	2(18.1)	3 (25)
ANA positive	4(50)	7 (58.3)
ILAR categories		
Systemic arthritis	9 (81.8)	1(8.3)
Oligoarthritis	0 (0)	2(16.6)
Polyarthritis	2 (18.1)	5 (41.6)
Enthesitis-related arthritis	0(0)	4 (33.3)
Prior use of drugs		
NSAID	11(100)	12(100)
Methotrexate	10(90)	12(100)
Azathioprine	7(63)	5 (41.6)
Sulfasalazine	1 (9)	3 (25)
Prednisolone	10(90)	10(83)
Current use of drugs		
Methotrexate	10(90)	12(100)
Oral glucocorticoid	9 (81.8)	8 (66)

*Values are mean±SD, or number (%).

JIA = juvenile idiopathic arthritis; RF = rheumatoid factor; ANA = antinuclear antibodies; LAR =  International League of Associations for Rheumatology.

NSAID = Non-steroidal anti-inflammatory drug.

### Longitudinal change in complete blood counts, CRP and lipid level

**Figure 1:**
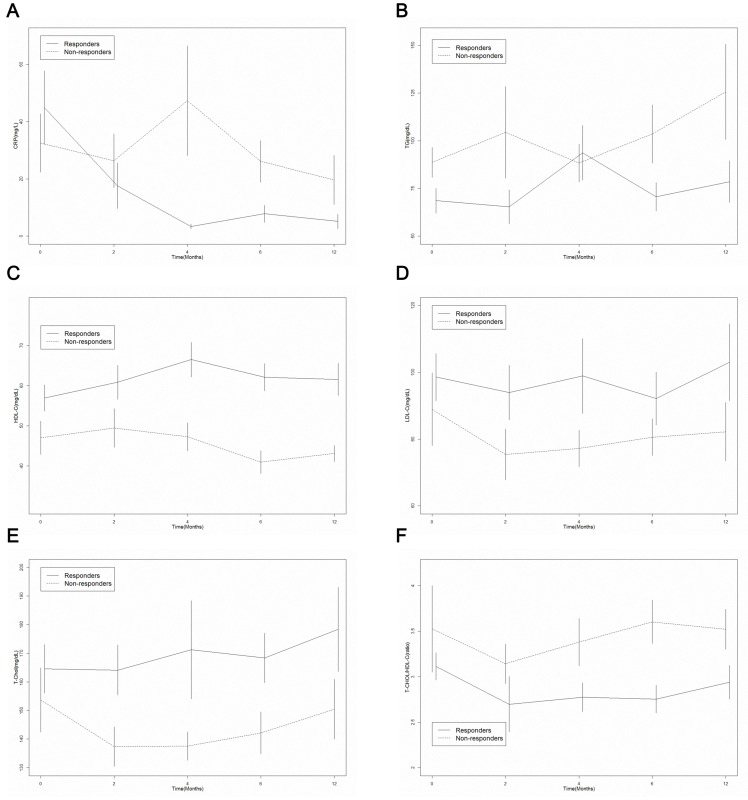
The change of inflammation marker and lipid profiles of JIA patients after etanercept treatment. (A) CRP (B) TG level significantly decreased after etanercept treatment in the inactive patients than active patient.(C) HDL-C (D) LDL-C (E) T-Chol, and (F) T-Chol/HDL-C ratio significantly increased after etanercept treatment in the inactive patients than active patient. CRP = C-reactive protein; TG = triglycerides; HDL-C = high-density lipoprotein cholesterol; LDL-C = low-density lipoprotein cholesterol; T-Chol  = total cholesterol

After 12 months of etanercept treatment in total JIA patients, WBC counts, platelet and CRP levels had decreased gradually with statistically significant findings (p = 0.002, p = 0.006 and p = 0.006,respectively). The rise in Hemoglobin level was also significant after 12 months (11.1±1.8 to 12.3±1.3 g/dL, p = 0.002). The changes in TG,TC,HDL-C and LDL-C over time compared with baseline were not significant after 12 months (p = 0.062, p = 0.593, p = 0.493 and p = 0.740, respectively). Similar results were observed in the responders. An analysis of the data showed significant and progressive reduction in WBC counts, platelet and CRP levels during the 12-month follow-up period in the responders (p = 0.027 p = 0.024, p = 0.09; respectively) (Table 2). Among the non-responders, however, none of complete blood counts, CRP or lipid profile changed significantly (Table 3). The longitudinal dynamic changes in CRP, TG, HDL-C, LDL-C, TC, and TC/HDL-C ratio during the 12-month follow-up period are shown in Figure 1 which CRP and TG level significantly decreased after etanercept treatment in responders than non-responders. HDL-C, LDL-C, TC and atherogenic index significantly increased after etanercept treatment in responders than non-responders.

**Table 2:** Lipid profile and blood dynamic changes in responders.

Responders	Before Etanercept treatment	2 M after Etanercept treatment		4 M after Etanercept treatment		6 M after Etanercept treatment		12 M after Etanercept treatment	
	Mean±SD	Mean±SD	p-value	Mean±SD	p-value	Mean±SD	p-value	Mean±SD	p-value
Hb (g/dL)	11.5±1.5	11.7±1.1	0.297	12.1±0.9	0.017*	12.4±0.9	0.009*	12.8±0.7	0.002*
WBC (1000/uL)	12.4±4.9	10.8±5.6	0.143	9.3±2.8	0.049*	9.8±3.8	0.007*	8.8±2.7	0.024*
Platelet (10000/uL)	43.0±12.1	38.5±14.1	0.333	33.9±6.5	0.015*	35.5±9.5	0.009*	32.2±6.0	0.009*
HDL-C (mg/dl)	56.9±11.3	60.9±12.1	0.474	66.5±12.2	0.134	62.1±10.3	0.236	61.6±12.1	0.416
LDL-C (mg/dl)	98.6±24.4	94.0±23.1	0.573	98.9±31.8	0.531	92.2±23.9	0.980	103±34.6	0.703
TC (mg/dl)	164.6±29.4	164.1±26.1	0.727	171.2±51.4	0.360	168.3±26.0	0.304	178±44.2	0.260
TG(mg/dl)	68.6±22.4	65.4±25.2	0.490	93.7±43.1	0.126	70.7±22.2	0.810	78.6±32.9	0.165
CRP (mg/l)	44.9±44.5	17.6±26.4	0.091	3.4±2.7	0.019*	7.8±9.5	0.034*	5.1±8.0	0.027*

The p values were tested by paired-sample t test and nonparametric test. All the p values were tested with values from before etanercept treatment; *p<0.05, statistically significant.

CRP = C-reactive protein; HDL-C = high-density lipoprotein cholesterol; LDL-C = low-density lipoprotein cholesterol; TC = total cholesterol; TG = triglycerides.

**Table 3:** Lipid profile and blood dynamic changes in non-responders.

non-responders	Before Etanercept treatment	2 M after Etanercept treatment		4 M after Etanercept treatment		6 M after Etanercept treatment		12 M after Etanercept treatment	
	Mean±SD	Mean±SD	p-value	Mean±SD	p-value	Mean±SD	p-value	Mean±SD	p-value
Hb (g/dL)	10.8±2.1	10.9±1.6	0.790	11.0±1.4	0.611	11.3±1.8	0.358	11.8±1.5	0.118
WBC (1000/uL)	9.6±4.8	8.5±2.5	0.734	9.7±4.2	0.944	8.9±3.4	0.679	8.4±3.5	0.390
Platelet (10000/uL)	45.6±20.2	36.4±15.1	0.208	44.6±17.0	0.901	41.4±14.8	0.549	34.7±9.4	0.120
HDL-C (mg/dl)	47.0±13.1	49.5±12.8	0.730	47.2±10.6	0.719	40.9±9.5	0.275	43.1±6.7	0.452
LDL-C (mg/dl)	88.9±34.4	75.4±20.1	0.350	77.2±16.4	0.215	80.6±18.3	0.508	82.2±29.1	0.523
TC (mg/dl)	153.7±35.5	137.4±19.4	0.192	137.6±14.9	0.226	142.2±24.1	0.399	150.5±34.7	0.772
TG (mg/dl)	88.7±25.0	104.4±67.9	0.598	88.3±29.9	0.948	103.5±50.6	0.244	125.6±82.8	0.149
CRP (mg/l)	32.5±33.9	26.3±29.6	0.483	47.3±60.5	0.466	26.1±24.2	0.298	19.7±28.4	0.097

The p values were tested by paired-sample t test and nonparametric test. All the p values were tested with values from before etanercept treatment; CRP = C-reactive protein; HDL-C = high-density lipoprotein cholesterol; LDL-C = low-density lipoprotein cholesterol; TC = total cholesterol; TG = triglycerides.

### Comparison between responders versus non-responders during treatment

Compared to the responders and non-responders after GEE analysis, significantly higher mean levels of Hb (0.9172 g/dL, p = 0.0476), HDL-C (16.0381 mg/dL, p<0.0001) and TC (24.5882 mg/dL, p = 0.0206) were found in responders than in non-responders (Table 4). Significantly lower mean levels of TG (−26.6028 mg/dL, p = 0.0477) and TC/HDL-C ratio (−0.581, p = 0.0153) were also found in responders. The CRP level was lower (−13.9204 mg/L) and tend to decrease in responders, but it did not change significantly (p = 0.1373). Adjustment for all baseline characteristics (age, gender, and time) in the analysis had the similar results. Significantly higher mean levels of HDL-C (16.6861 mg/dL, p<0.0001), LDL-C (16.0705 mg/dL, p = 0.0447) and TC (26.5485 mg/dL, p = 0.0077) were found in responders (Table 5). Significantly lower mean levels of TG (−26.266 mg/dL, p = 0.0475) and TC/HDL-C ratio (−0.5967, p = 0.0045) were also noted in responders (Table 5).

**Table 4:** GEE analysis in responders versus non-responders during treatment (no adjust cofactor).

Outcome	Levels	B	Lower	Upper	p-value
Hb(g/dL)	R	0.9172	0.0096	1.8248	0.0476*
	N	0.0000	0.0000	0.0000	
WBC(1000/uL)	R	1.2502	−1.2640	3.7644	0.3298
	N	0.0000	0.0000	0.0000	
Platelet(10000/uL)	R	−39.7577	−119.0546	39.5391	0.3258
	N	0.0000	0.0000	0.0000	
HDL-C(mg/dl)	R	16.0381	9.3458	22.7305	<0.0001*
	N	0.0000	0.0000	0.0000	
LDL-C(mg/dl)	R	16.0991	−1.5544	33.7526	0.0739
	N	0.0000	0.0000	0.0000	
TC/HDL-C ratio	R	−0.5816	−1.0518	−0.1114	0.0153*
	N	0.0000	0.0000	0.0000	
LDL-L/HDL-C ratio	R	−0.2443	−0.6507	0.1622	0.2388
	N	0.0000	0.0000	0.0000	
TC(mg/dl)	R	24.5882	3.7782	45.3981	0.0206*
	N	0.0000	0.0000	0.0000	
TG(mg/dl)	R	−26.6028	−52.9315	−0.2741	0.0477*
	N	0.0000	0.0000	0.0000	
CRP(mg/l)	R	−13.9204	−31.8040	3.9633	0.1271
	N	0.0000	0.0000	0.0000	

*p<0.05, statistically significant.

R: responders; N: non- responders; CRP = C-reactive protein; HDL-C = high-density lipoprotein cholesterol; LDL-C = low-density lipoprotein cholesterol; TC = total cholesterol; TG = triglycerides.

**Table 5:** GEE analysis in responders versus non-responders during treatment (adjusted for age, gender and time cofactors).

Outcome	Levels	B	Lower	Upper	p-value
Hb(g/dL)	R	0.6181	−0.1460	1.3823	0.1129
	N	0.0000	0.0000	0.0000	
WBC(1000/uL)	R	1.3234	−1.2023	3.8490	0.3044
	N	0.0000	0.0000	0.0000	
Platelet(10000/uL)	R	−27.0561	−100.6331	46.5210	0.4711
	N	0.0000	0.0000	0.0000	
HDL-C(mg/dl)	R	16.6861	10.2569	23.1154	<0.0001*
	N	0.0000	0.0000	0.0000	
LDL-C(mg/dl)	R	16.0705	0.3824	31.7586	0.0447*
	N	0.0000	0.0000	0.0000	
TC/HDL-C ratio	R	−0.5967	−1.0079	−0.1854	0.0045*
	N	0.0000	0.0000	0.0000	
LDL-C/HDL-C ratio	R	−0.2684	−0.6314	0.0947	0.1474
	N	0.0000	0.0000	0.0000	
TC(mg/dl)	R	26.5485	7.0154	46.0816	0.0077*
	N	0.0000	0.0000	0.0000	
TG(mg/dl)	R	−26.2666	−52.2437	−0.2895	0.0475*
	N	0.0000	0.0000	0.0000	
CRP(mg/l)	R	−13.8714	−32.1664	4.4235	0.1373
	N	0.0000	0.0000	0.0000	

*p<0.05, statistically significant.

R: responders; N:non- responders; CRP = C-reactive protein; HDL-C = high-density lipoprotein cholesterol; LDL-C = low-density lipoprotein cholesterol; TC = total cholesterol; TG = triglycerides.

## Discussion

Cardiovascular disease is the leading cause of mortality in rheumatic patients, and the most important factors are atherosclerosis and inflammatory status. However, this is seldom discussed with regards to JIA patients. We used longitudinal follow-up of JIA patients according to clinical symptoms and the dynamic change in complete blood counts, inflammatory mediators and lipid levels. Our data shows for the first time that the favorable effect TNFα inhibitor treatment has on lipid only applies to responders of JIA patients. We observed a close relationship between JIA disease activity and lipid levels consistent with previous findings. We found that the higher inflammatory state in JIA patients is associated with the risk of dyslipidemia. However, unlike adults, children and adolescents have fewer chronic diseases and traditional cardiovascular risk factors, so this JIA cohort presents with a baseline to further investigate the role of chronic inflammatory arthritis in the pathogenesis of dyslipidemia and the hemodynamic change after etanercept treatment.

Although the relationship between dyslipidemia and inflammatory conditions of rheumatoid arthritis in adults has been mentioned in many studies, results remain still controversial[1], [11], [21]. Moreover, only a few published studies specifically discuss this subject with regards to JIA patients, and several have had inconsistent results. Ilowite et al were the first to describe an altered lipoprotein profile in JIA patients that was characterized by decreased HDL levels and increased TG and VLDL levels[15]. Dyslipidemia with decreased HDL-C levels and elevated levels of low-density lipoprotein cholesterol, triglycerides, and total cholesterol were identified with polyarticular juvenile idiopathic arthritis by Marangoni et al [22]: however, they presented that dyslipidemia with decreased HDL-C was not affected by disease activity or therapy. Another two studies showed an insignificant diminution of LDL-C in patients with active disease when compared to either patients without active disease or to controls [23], [24]. However, the results could be biased due to factors that were not the controlled and the lack of longitudinal study design. Disease severity was also different and it is an important issue that influences lipid profile level. In two other studies published by Bakkaloglu et al [23] and Tselepis et al [25], similar pro-atherogenic patterns of serum lipids closely related to disease activity in JIA patients have already been described. Interestingly, these abnormalities were compatible to our findings. In our study, when comparing responders and non-responders, higher HDL-C, TC and lower TG and TC/HDL-C ratio were found in responders. A study by Vermont et al showed reduced cholesterol level in children upon admission with severe meningococcal sepsis and were completely normal 1−3 months after admission were prescribed [26]. And they also found that TC, HDL-C, and LDL-C levels on admission are inversely associated with disease severity. Our finding also showed that TC, HDL-C, and LDL-C were higher in responders than in non-responders after etanercept treatment. Therefore, we suggest that disease severity was associated with atherogenic index (TC/HDL-C ratio) and inversely associated with TC, HDL-C, and LDL-C levels.

To date, several studies have examined the mechanisms of why there is a correlation between inflammation and lipid profiles in JIA patients, but a definitive understanding of this correlation has not yet been established. Cytokine induced activation of the reticuloendothelial system is hypothesis by Shen C-C, et al [27]. A significant correlation is noted between clinical variables of disease severity with levels of interleukin-6 and tumor necrosis factor-α. As reported in adults with chronic arthritis, treatment with biologics targeting tumor necrosis factor-α receptor decreased all parameters of inflammation and increase HDL-C and TC levels significantly [21], [28]. In non-responders group, the majority were systemic type JIA which seems to be less effective when treating with TNF alpha antagonist than other categories. With understanding on disease pathogenesis, biologics blocking IL-1 and IL-6 played more effective treatment role on systemic type JIA. This is one of the explanations that lipid profiles would not respond dominantly either in our study. Other biologic agents have also been shown to affect lipid profiles. Tocilizumab (TCZ), which inhibits the proinflammatory cytokine IL-6 binding to its receptors, is associated with decreases in inflammatory markers [29]. In the present study, we also find significantly higher HDL-C and lower TG and TC/HDL-C in responders (Table 5). Therefore, based on the present study, we hypothesize that lipid abnormalities correlate with disease activity which could be associated with an increased risk of cardiovascular mortality and morbidity in active JIA, however, this could be improved after adequate anti-inflammatory intervention.

A number of limitations with regards to this study should be considered. Firstly, the relatively small sample size in this study may raise some questions about the statistical power of these findings. Secondly, all baseline variables were considered as potential confounders; however, we were not able to exclude confounding by unmeasured factors - for example, smoking, exercise, steroid or non-steroidal anti-inflammatory drug use and so on. Thirdly, because of the longitudinal cohort design of the study, missing data may have led to bias. Therefore, we attempted to use GEE analysis, as it adjusts for dependency of several measurements within one subject and is capable of dealing with unequally spaced time intervals and with missing data [30]. Finally, atherosclerosis is a chronic and multifactorial process in which long-term changes in the lipid profile will affect CV risk [31]. We considered that JIA is a chronic inflammation disease which can cause hyperlipidemia. We only followed up our patients for 1 year, whereas a longer period of time may be required to address long-term outcomeIn the present study, all patients were at an active stage in their disease in the beginning and traditional treatment had already failed. In other words, they had severe, persistent JIA and no effective traditional treatment was available, as reflected by the long duration of disease, large number of active joints and higher level of inflammatory status. Half of the patients (12/23) had good response to etanercept after 12 months of treatment. The results suggest that etanercept provided a good response to some JIA patients, but the remaining JIA patients still needed to use other treatments to control disease activity.

In conclusion, we have found that active JIA was associated with pro-atherogenic patterns of serum lipid profiles. Disease severity was associated with atherogenic index (TC/HDL-C ratio) and inversely associated with TC, HDL-C, and LDL-C levels. By using TNF- α receptor blocking agent(anti-inflammatory drug), the aberrant lipid profiles were improved by of the control of inflammation status, which may in turn reduce the risk of CVD in non-systemic type JIA. Additional prospective long- term study is needed to comprehensively investigate the role of inflammation and the impact of biologic agents on lipid levels and CV outcomes in patients with JIA.
